# Reproducible Analysis Pipeline for Data Streams: Open-Source Software to Process Data Collected With Mobile Devices

**DOI:** 10.3389/fdgth.2021.769823

**Published:** 2021-11-18

**Authors:** Julio Vega, Meng Li, Kwesi Aguillera, Nikunj Goel, Echhit Joshi, Kirtiraj Khandekar, Krina C. Durica, Abhineeth R. Kunta, Carissa A. Low

**Affiliations:** Department of Medicine, University of Pittsburgh, Pittsburgh, PA, United States

**Keywords:** digital health, digital phenotyping, mobile sensing, smartphone, wearable, digital biomarkers

## Abstract

Smartphone and wearable devices are widely used in behavioral and clinical research to collect longitudinal data that, along with ground truth data, are used to create models of human behavior. Mobile sensing researchers often program data processing and analysis code from scratch even though many research teams collect data from similar mobile sensors, platforms, and devices. This leads to significant inefficiency in not being able to replicate and build on others' work, inconsistency in quality of code and results, and lack of transparency when code is not shared alongside publications. We provide an overview of Reproducible Analysis Pipeline for Data Streams (RAPIDS), a reproducible pipeline to standardize the preprocessing, feature extraction, analysis, visualization, and reporting of data streams coming from mobile sensors. RAPIDS is formed by a group of R and Python scripts that are executed on top of reproducible virtual environments, orchestrated by a workflow management system, and organized following a consistent file structure for data science projects. We share open source, documented, extensible and tested code to preprocess, extract, and visualize behavioral features from data collected with any Android or iOS smartphone sensing app as well as Fitbit and Empatica wearable devices. RAPIDS allows researchers to process mobile sensor data in a rigorous and reproducible way. This saves time and effort during the data analysis phase of a project and facilitates sharing analysis workflows alongside publications.

## Introduction

Researchers in computer science, behavioral science, medicine, and other fields are increasingly harnessing data collected from smartphone sensors and wearable devices like smartwatches and activity bands to passively monitor people's activities and environment as they go about their daily lives.

Raw or preprocessed mobile sensor data (e.g., smartphone accelerometer logs or Fitbit step counts) collected over time are usually further manipulated to extract more meaningful behavioral features, such as number of incoming calls, minutes spent at home, or number of screen unlocks that are then used to create models of risk prediction or detection (1). If validated, these features have the potential to become behavioral phenotypes (2) or digital biomarkers (3–5). Because these data can be collected passively and prospectively with minimal participant burden, this approach holds considerable promise for risk screening, remote clinical monitoring, and personalized just-in-time interventions (6). For example, mobile sensor data features can be analyzed to discriminate between people such as depressed and non-depressed individuals (7–9), to detect or predict significant events such as an increase in symptoms during chemotherapy (10), or to explore and explain clinical or behavioral processes (11).

While available platforms and devices for mobile research data collection have increased in recent years, software tools to help researchers manage and make sense of mobile data in rigorous and reproducible ways remain less common. This paper describes RAPIDS, a research software tool that aims to address this gap in the scientific process and literature. Behavioral feature extraction from mobile sensor data is an essential but time-consuming and nuanced task that needs to consider problems like missing data, data format differences between and across device manufacturers and platforms, time granularity at which the data is analyzed, participants' time zones, etc. As a result, the outcome of data analysis can be inconsistent within and across teams, and the code's quality can vary. Furthermore, code is often not shared alongside publications; when it is, it might not be stored on a version control system and most of the time there is no guarantee the development environment can be replicated as programming languages and libraries are updated.

For example, we reviewed the first 200 results of a Google Scholar search carried out in March 2021 with the keywords “smartphone wearable digital phenotype biomarker feature” and identified 31 publications that collected smartphone or wearable data to extract behavioral features. These works processed data from different sources including accelerometers, light sensors, screen events, photoplethysmography measurement of heart rate, keyboard strokes, location and others, within varied time segments (windows) such as 1 min, 15 min, 6 h, 24 h, and 7 days (12–20). Among these papers, only four (21–24) released the source code of their data processing approaches or offered to provide code on-demand while the rest provided various levels of detail that do not guarantee their results can be replicated. Additionally, for most papers it was not clear if they had re-used code created by themselves or others or implemented their code from scratch. Although this is a convenience sample, we expect this ratio reflects the state of this growing literature as the same problem has been observed in other fields (25–31).

Given that “software must be recognized as an important output of scholarly research” (32), it is critical to develop shared resources to improve the rigor and reproducibility of mobile sensing work that supports and accelerates research in this new and rapidly growing field. Such resources would enable researchers to reproduce or extend previous findings with minimal duplication of effort and with full transparency of the many decisions and assumptions underlying the extensive data cleaning and processing required to translate mobile sensor data into meaningful and actionable signals.

Recently several tools have been created to alleviate some of the aforementioned mobile data processing and analysis problems. The Digital Biomarker Discovery Pipeline (DBDP) (5) computes features and provides statistical and machine learning analysis modules to predict health outcomes, but supports only wearable device data. The “Health Outcomes through Positive Engagement and Self-Empowerment” (HOPES) (33) platform extended the Beiwe ecosystem (34) and can process Android, iOS as well as Fitbit data collected with their platform, but is not publicly available yet. Forest is a Python library that as of August 2021 can summarize location, calls, and survey data collected with Beiwe's smartphone applications (35). Doryab et al. (36) provide Python scripts upon request to extract behavioral features from Fitbit devices and smartphone data logged by the AWARE Framework (37). Finally, the MD2K project (38) has a data analysis library that developers can use to extract behavioral features from data collected with their Android smartphone sensing platform and the MotionSenseHRV wearable.

To build on these existing tools, our team created RAPIDS to support a broader range of smartphone sensing applications and wearable devices and to encourage transparency and open science in mobile sensing research. The purpose of RAPIDS is to improve the rigor and efficiency of mobile sensing data analysis by addressing two problems. First, RAPIDS targets the time-consuming and laborious nature of this mobile sensing data processing and analysis by providing a modular, efficient, tested, and scalable software platform that researchers can use to reduce the time and effort required to extract new and existent behavioral features, visualize mobile data, and organize data modeling workflows. Second, RAPIDS targets the significant variability in how mobile sensor data is produced across teams, individuals, and time by relying on open algorithms and software packages that standardize data processing and analyses as well as on open discussions, documentation, and software distribution tools that support code sharing, open science, and reproducibility.

Even though this manuscript is neither an empirical paper nor a complete technical reference of RAPIDS, we aim to provide mobile health researchers with an overview of its functionality so they can decide whether to use RAPIDS to support their sensor data processing and analysis. For example, let us assume that a group of researchers want to develop a model to detect momentary stress levels in adults using mobile sensors for use in a future trial delivering a mobile stress reduction intervention at times of high stress. They recruit 100 participants living in two different time zones that, for 12 weeks, self-report their stress every 3 h, collect continuous location and event-based screen smartphone data with the AWARE framework and log heart rate data every minute using a Fitbit device. One of the researchers' goals is to train a machine learning classification model to predict momentary stress. In the rest of this paper, we describe the functionality that would allow these hypothetical investigators to process their participant sensor data to extract behavioral features and create plots of data compliance in a reproducible, extensible, and robust way. After this step, researchers can use such features to create the desired statistical and machine learning models in their favorite programming language. Real world deployments of RAPIDS have been used for predicting depression symptoms (9, 39), perioperative symptom burden estimation (40), creating individual signatures linking brain, behavior, and mood (41) and as a part of a machine learning pipeline for monitoring and forecasting mobile health data (42).

## Methods

**Figure 1:**
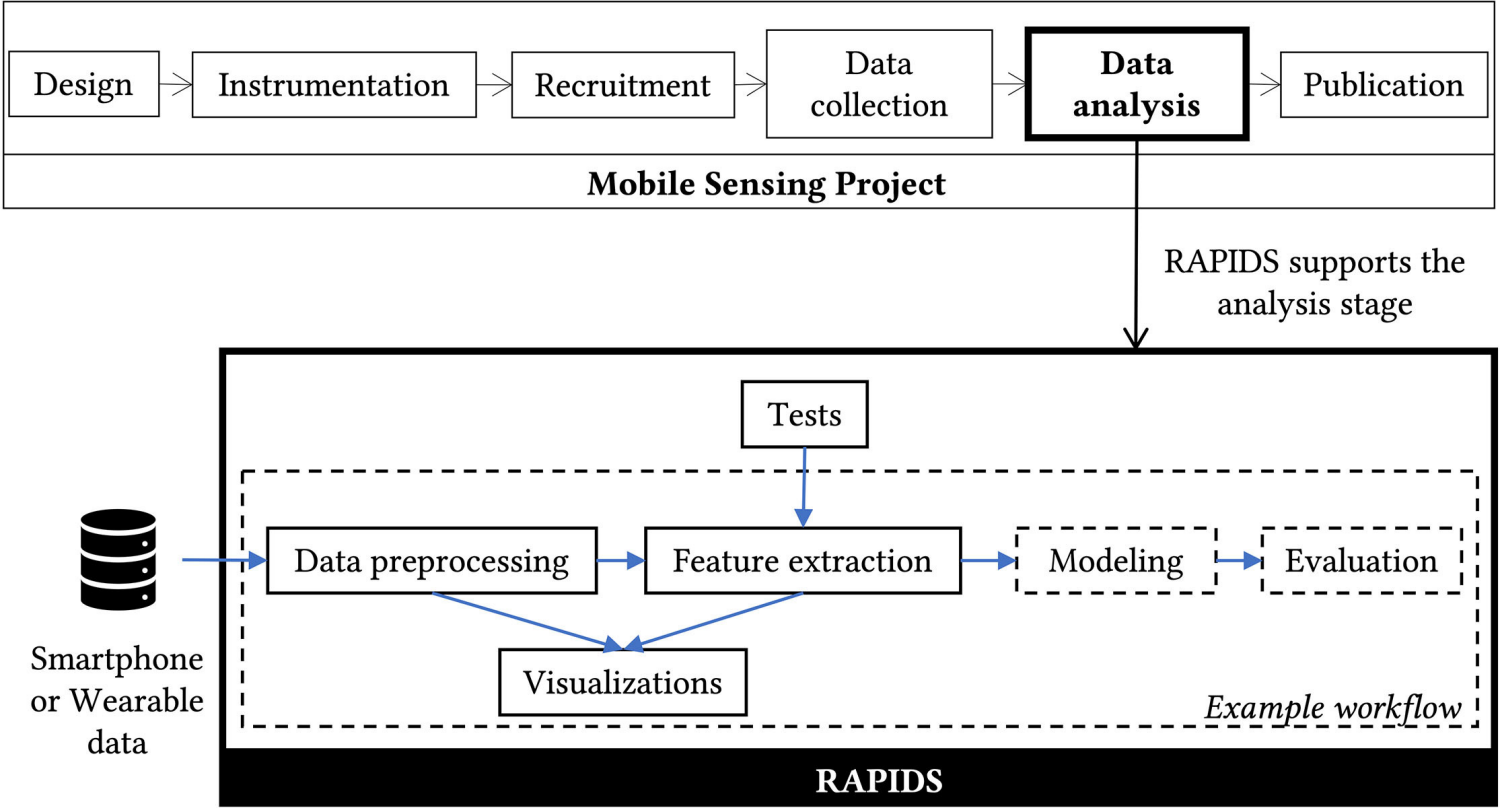
RAPIDS supports researchers during the data analysis phase of a mobile sensing project. RAPIDS scripts can be categorized by purpose, those with a continuous border are reusable by other projects while those with a dashed border are provided as an example so other researchers can implement their own analysis.

A mobile sensing research project has roughly the following stages: design, instrumentation, recruitment, data collection, data analysis, and publication. Researchers can use RAPIDS during data analysis if they collected Android or iOS smartphone data, or wearable data using Fitbit and Empatica devices (see Figure 1).

RAPIDS is an open-source collection of Python and R scripts that are executed by the Snakemake workflow manager (43) and organized based on the cookie cutter data science project structure (44). Its source code is published in GitHub under a GNU Affero General Public License v3.0 and the project has adopted the Contributor Covenant code of conduct (45). Installation, configuration, usage, and development documentation is available online (46). RAPIDS can be installed in Windows, MacOS and Linux using our Docker container or natively in the last two platforms using Python and R virtual environments.

RAPIDS provides modules, which we call *providers*, for behavioral feature extraction and data visualization. RAPIDS can compute 5 plots and 407 behavioral features from 15 smartphone sensors, 4 Fitbit sensors through 20 providers (see Table 1). Behavioral features are grouped per sensor, per participant and per study on CSV files that can be used as input for statistical or machine learning models. Although RAPIDS does not yet provide modules to create statistical or machine learning models, it does provide an analysis workflow example that guides users though the implementation of their own models while taking advantage of RAPIDS' capabilities. Other researchers can also extend RAPIDS to support their own behavioral features, mobile sensing apps, and data containers.

**Table 1:** Number of behavioral features supported in RAPIDS.

**Sensor**	**Providers**	**Platform**	**Features**	**Time segments**
Accelerometer	2	Android/iOS	11	All
Activity recognition	1	Android/iOS	6	All
Applications crashes	0	Android	0	-
Applications foreground	1	Android	4	All
Applications notifications	0	Android	0	-
Battery	1	Android/iOS	6	All
Bluetooth	2	Android	30	All
Calls (incoming)	1	Android/iOS	12	All
Calls (outgoing)	1	Android/iOS	12	All
Calls (missed)	1	Android/iOS	5	All
Conversation	1	Android/iOS	30	All
Data yield	1	Android/iOS	2	All
Keyboard	1	Android	10	All
Light	1	Android	6	All
Locations	2	Android/iOS	34	All/N days
SMS (sent & received)	1	Android	10	All
Screen	1	Android/iOS	7	All
Wi-Fi connected	1	Android/iOS	3	All
Wi-Fi visible	1	Android	3	All
Data yield	1	Fitbit	2	All
Calories intraday	1	Fitbit	22	All
Heart rate summary	1	Fitbit	37	N days
Heart rate intraday	1	Fitbit	13	All
Steps summary	1	Fitbit	5	N days
Steps intraday	1	Fitbit	17	All
Sleep summary	1	Fitbit	36	N days
Sleep intraday	2	Fitbit	34	All/N days
Accelerometer	1	Empatica	5	All
Heart rate	1	Empatica	9	All
Peripheral skin temperature	1	Empatica	9	All
Electrodermal activity	1	Empatica	9	All
Blood volume pulse	1	Empatica	9	All
Inter beat interval	1	Empatica	9	All

Consistent with open science practices, the development of RAPIDS is community-driven and we are transparent about the algorithms and assumptions we have made in our processing computer code, encouraging researchers to participate in such conversations and to modify RAPIDS code as needed to suit their needs and research questions via GitHub issues (47). So far other researchers have shared behavioral features for accelerometer, Bluetooth, used applications, and location data (36, 48–50). We invite others to contribute with their work as RAPIDS has the potential to allow other members of the community to reuse it while keeping citations and authorship.

## Results

### RAPIDS Capabilities

RAPIDS implements novel capabilities to support certain aspects of data processing, open-source development, and reproducibility of mobile sensing projects.

#### Supported Devices and Sensors

RAPIDS can compute behavioral features for calories, heart rate, sleep, and steps Fitbit sensors; accelerometer, heart rate, skin temperature, electrodermal activity, blood volume pulse, and inter beat interval Empatica sensors; and the following smartphone sensors: accelerometer, activity recognition, application notifications, used applications, application crashes, application logs, battery, Bluetooth, incoming calls, outgoing calls, missed calls, conversations, keyboard, light, locations, sent and received messages (SMS), screen, visible Wi-Fi access points and connected Wi-Fi access points. As of August 2021, RAPIDS can process smartphone data logged with the AWARE Framework and stored in CSV files, MySQL, and InfluxDB databases but researchers can bring support for any other storage medium and Android or iOS mobile sensing applications.

#### Flexible Time Segments

In mobile sensing research, behavioral features are usually extracted within specific time windows that aim to summarize human activities at a specific time granularity, for example every hour or day. RAPIDS provides users with three types of flexible time segments that enable them to compute features that have the potential to adapt to many sensing study designs. Frequency segments represent repetitive windows of the same length on every day of a study; e.g., 5-min windows starting from midnight, which could be useful for momentary stress or sleep classification. Periodic segments represent periods of any length that can start at any time of every day or on specific days of the week, month, quarter, or year. These segments are useful to create popular extraction periods that span mornings, weekends, weekdays, weeks, overnights, and others, allowing researchers to examine how behaviors vary over these periods. Finally, event segments represent periods of any length that can start before, on or after specific events of interest as defined by each study. These segments are meant to quantify human behavior around events like ecological momentary assessments or adverse health incidents like migraines or drinking episodes.

#### Flexible Time Zones

RAPIDS automatically adjusts participants' sensor data to one or more time zones on which it was originally collected. This is relevant for studies that recruited people living across different time zones or participants that traveled during their enrollment. Researchers do not have to deal with daylight saving changes or date arithmetic. Flexible time zones and time segments can process sensor data streams that are supposed to be interpreted as “episodes” spanning multiple hours or days and need to be segmented like screen or sleep. They also ensure features extracted from different sensors and devices are aligned based on their creation date and time. For example, if the user is extracting daily features from smartphone and Empatica data, these features will automatically be indexed by the midnight-to-midnight windows where data is present for either or both devices.

#### Device Study Management

RAPIDS can merge data from multiple smartphone or wearable devices that were used by the same participant. Data merging is a common problem when people carry more than one device or switch devices during a study. RAPIDS also provides plots and estimations of smartphone and Fitbit data yield that represent monitored and unmonitored periods due to factors like data synchronization problems, mobile app crashes or a discharged battery. Researchers can use this information to discard time segments with insufficient data. For example, any inferences made on a day with only 1 h of mobile data available can be considered less valid than inferences made on days with 24 h of data; each research team can decide where the validity threshold lies.

#### Modular, Scalable, and Transparent Workflows

RAPIDS uses the workflow manager Snakemake to organize analysis pipelines into contained, ready-to-use, scalable, auditable steps. These steps produce the different behavioral features and plots RAPIDS supports, can be configured using plain text files and do not require researchers to produce any computer code. In RAPIDS, every sensor for every participant goes through the exact same processing in isolated steps with input and output files that can be inspected at any time. This in turn means that the workflow is efficient because an analysis step is only executed when its input or parameters change and, when this happens, any dependent step is automatically re-computed. For example, if the accelerometer data for participant A is updated, only features for that sensor and that participant will be updated, while features for any other sensor or person will keep the most recent results. Finally, this step-based structure allows researchers to execute their analysis workflows over multiple CPU cores or computer cluster nodes without modifying RAPIDS' code.

#### Reproducible Programming Environments

All the scripts in RAPIDS run on top of isolated R and Python virtual environments which means that when a RAPIDS workflow is shared online along with a research paper, it can be reinstalled and rerun using the same libraries and producing the same results that the authors intended regardless of any software updates to the libraries the workflow relies on.

#### Tests

We have also implemented tests for 17 out of the 25 mobile sensors we support to verify that our code produces correct results under different scenarios, and we are constantly adding more tests and scenarios.

#### Web Documentation

RAPIDS is supported by thorough and consistent online documentation that includes installation, configuration, and usage instructions, a description of the supported behavioral features and data streams, tutorials to add new ones, common troubleshooting, available test cases, code of conduct, and citation guidance (46).

#### Data Visualizations

RAPIDS provides five interactive plots for data exploration. These include histogram and heatmaps for data yield per study, participant, sensor, and time segment, as well as a correlation matrix for extracted behavioral features. New plots can be added by the community.

#### Data Analysis in a RAPIDS Workflow

Even though the bulk of RAPIDS' current functionality is related to the computation of behavioral features, we recommend RAPIDS as a complementary tool to create a mobile data analysis workflow. This is because RAPIDS capabilities allow researchers to divide an analysis workflow into small parts that can be audited, shared in an online repository, reproduced in other computers, and understood by other people as they follow a familiar and consistent structure. To create analysis workflows in RAPIDS, researchers can still use any data manipulation tools, editors, libraries, or languages they are already familiar with. RAPIDS is meant to be the destination of analysis code developed in interactive notebooks or stand-alone scripts. The idea is that when it is time to publish a piece of research, a RAPIDS workflow can be shared in a public repository as is, making it easy for other teams or collaborators to replicate and extend mobile sensing results. We describe an example workflow in our online documentation.

### Preliminary Usability Evidence

To obtain preliminary evidence of the usability, utility, and value of RAPIDS for mobile sensing researchers, we surveyed five early adopters of RAPIDS from three Universities in the USA and one in Finland. Two of them are PhD. candidates and three are research assistants with a background in Computer Science or similar that used RAPIDS to extract behavioral features.

They completed the System Usability Scale (SUS) with an average score of 73.5 corresponding to a Sauro-Lewis Curved Grading Scale Grade (51) of B-; 0.6 points below the mean usability score range for Internal Productivity Software (IPS). Based on the SUS' Item benchmarks (52) targeting a score of 76.7 (mean score for IPS), items 1, 2, 3, 5, 7, and 8 represented an above average experience while items 4, 6, 9, and 10 a below average experience. These results indicate that RAPIDS' complexity, ease of use, and functionality integration are good, but users perceived some inconsistency and a relevant learning curve which affected how confident they were using the system. We expect that future documentation updates based on our users' feedback and alternative didactic resources like video tutorials will support users' learning process.

Despite the initial effort required to get familiar with RAPIDS, our users reported significant benefits. They perceived RAPIDS made their feature engineering process two, four, and up to six times faster with net savings of 1, 2.5, 8, 15, and 100 hours, acknowledging that they would have had to implement their own computer scripts if RAPIDS was not available. Additionally, everyone thought that RAPIDS makes the reproducibility of a mobile sensing project “much better,” that it makes them “somewhat more” or “much more” confident in their own and other's results, and that it “somewhat more” or “much more” improves their ability to add new mobile devices or participants to their analysis. Finally, on a scale from 0 to 100 with 0 being “not at all likely” and 100 “extremely likely,” users reported an average score of 89 (range 80–100) on their likeliness to use RAPIDS again and 91 (range 85–100) to recommend RAPIDS to a colleague. Everyone agreed or strongly agreed that RAPIDS “could advance the field of mobile sensing research.”

Overall, our participants' answers suggest that RAPIDS provides data analysis functionalities for mobile sensing projects that reduce users' effort and are easy to use, are faster than implementing your own analysis code, and represent distinct contributions to the mobile data analysis landscape.

### RAPIDS Behavioral Features

RAPIDS organizes behavioral features by sensor and by provider. A provider is an R or Python feature extraction script implemented by a group of authors for a particular mobile sensor. Most features are implemented by our team (provider RAPIDS) but we also include code created by other researchers (in our documentation we ask users to cite these other works as well as RAPIDS). Some sensors are only available for specific smartphone platforms due to their own restrictions, e.g., at this time it is not possible to collect app usage data in iOS similar to what can be collected in Android. We recommend the reader checks the latest online documentation as new features will be added in the future. Researchers can choose to extract as many features as needed for their research, whether they plan to use meaningful summary features like longest sedentary bout duration or percentage of time spent at home in traditional statistical analyses or to use a larger array of features in machine learning models aimed at detecting or predicting changing health states.

Our online documentation lists considerations for each sensor that RAPIDS takes into account to compute behavioral features. Some are inherent to the smartphone platforms (Android or iOS) while others are introduced by the sensing mobile applications. RAPIDS can also be extended to support any mobile sensing app, but it was initially built with the AWARE Framework in mind. Thus, some of the listed sensors might not be available in other smartphone applications.

## Discussion

RAPIDS is an open-source pipeline designed to save researchers time and effort through documented, reproducible, consistent, efficient, and parallel processing of mobile sensor data. As of August 2021, it can extract 407 behavioral features from smartphones, Fitbit, and Empatica devices and provides five data visualization plots. Users do not need to write any computer code to compute these features within time segments of any length that start around specific days or events. At the same time, researchers can implement new features and add support for new sensing devices or platforms like “Effortless Assessment of Risk States” (EARS) (53), “Learn Assess Manage and Prevent” (LAMP) (54), or Beiwe (34). RAPIDS is also suggested as a tool to organize and share analysis workflows that would provide future readers a familiar, transparent, and reproducible analysis environment. We hope all these functionalities will encourage scientists to share their work and therefore allow third parties the ability to compare, reuse, and build upon the methods and results of mobile behavioral sensing studies. If the community adopts RAPIDS or a similar tool, it has the potential to unify the behavioral features used in research and accelerate progress in the field. Although there is a risk that uncovered bugs in RAPIDS could systematically bias any projects using it in the future, we believe that the transparency that comes with an open-source project, community engagement, and our efforts toward testing our code will help mitigate this risk.

In the future, the core development team of RAPIDS and we hope the community will add new functionality to this project. We expect to support combinatorial features that mix data from multiple sensors. Data cleaning modules based on packages like vtreat (55) are a work in progress, as well as integration with data testing libraries like Great Expectations (56). Additionally, we plan to support other wearable devices like the Oura Ring (57) or continuous glucose monitors. Finally, we hope to contribute new visualizations and reports for data quality control and exploration. Given the time constraints of our team, we expect that most of these improvements will come on an as-needed basis. Still, we encourage interested colleagues to consider contributing or to get in touch to discuss priorities that benefit the research community.

## Conclusions

We presented RAPIDS, an open-source, reproducible, documented, extensible, and tested pipeline that ships with behavioral features and plots that can be extracted from data collected with Android and iOS smartphones as well as Fitbit and Empatica devices. We also provided a workflow example that other researchers can follow to structure their own data analysis pipelines within RAPIDS that can be shared online along with research publications. RAPIDS capabilities support data processing, development, and reproducibility of mobile sensing projects and enable other scientists to replicate or extend previous results with minimal duplication effort and complete transparency.

## Data Availability Statement

Publicly available datasets were analyzed in this study. This data can be found at: https://www.rapids.science and https://github.com/carissalow/rapids.

## Author Contributions

All authors listed have made a substantial, direct and intellectual contribution to the work, and approved it for publication.

## Funding

This work was supported in part by the National Cancer Institute (K07CA204380, R37CA242545, and P30CA047904).

## Conflict of Interest

The authors declare that the research was conducted in the absence of any commercial or financial relationships that could be construed as a potential conflict of interest.

## Publisher's Note

All claims expressed in this article are solely those of the authors and do not necessarily represent those of their affiliated organizations, or those of the publisher, the editors and the reviewers. Any product that may be evaluated in this article, or claim that may be made by its manufacturer, is not guaranteed or endorsed by the publisher.
